# Anti-migraine medications safety during pregnancy in the US

**DOI:** 10.3389/fphar.2024.1481378

**Published:** 2024-12-17

**Authors:** A. Bérard, S. Strom, D. Albrecht, S. Kori

**Affiliations:** ^1^ Faculty of Pharmacy, University of Montreal, Montreal, Canada; ^2^ Research Center, CHU Ste-Justine, Montreal, QC, Canada; ^3^ Faculty of Medicine, Université Claude Bernard Lyon 1, Lyon, France; ^4^ Satsuma Pharmaceuticals, Inc., San Francisco, CA, United States; ^5^ Independent Consultant, Los Angeles, CA, United States

**Keywords:** migraine, pregnancy, spontaneous abortion, major congenital malformations, prematurity, low birth weight, medications, US

## Abstract

**Background:**

Specific antimigraine medications (dihydroergotamine (DHE), triptans) have been associated with adverse pregnancy outcomes in individual studies but lack of consensus remains.

**Objectives:**

Quantify the risk of prematurity, low birth weight (LBW), major congenital malformations (MCM), and spontaneous abortions (SA) associated with gestational use of DHE or triptans in a privately insured cohort of pregnant women in the US.

**Methods:**

We conducted a cohort study within the US Merative MarketScan Research Database (2011–2021), composed of a nationally representative sample of patients with employer-provided health insurance. Four independent analyses were conducted to assess the risk of 1) prematurity (<37 weeks of gestation), 2) LBW (birth weight <2,500 g), 3) MCM, and 4) clinically detected SA. Exposure was defined dichotomously as use of DHE or triptan during pregnancy. Generalized estimation equations (GEE) were built to quantify the associations taking into account potential confounders including maternal migraine.

**Results:**

Overall, 767,994 pregnant women met eligibility criteria and were included in the analyses on prematurity, LBW, and MCM; 11,121 cases of SA were identified and analyzed. One hundred and eighty-nine (189 (0.02%)) were exposed to DHE (all in the first trimester), and 4,309 (0.56%) to triptans. Adjusting for potential confounders including maternal migraine, DHE was not associated with a statistically significant risk of prematurity (adjusted RR (aRR) 1.17, 95%CI 0.14, 9.74), LBW (aRR 7.76, 95%CI 0.99, 60.83), MCM (aRR 2.27, 95%CI 0.97, 5.29), or SA (aOR 3.19, 95%CI 0.98, 10.38); DHE was associated with an increased risk of septal defects. All estimates showed increased risk but were unstable. Similarly, triptan use was not associated with any of the studied outcomes.

**Discussions and Conclusions:**

After considering maternal migraine and other potential confounders, DHE (first trimester) and triptan exposure during pregnancy were not statistically significantly associated with an increased risk for prematurity, LBW, MCM, or SA. Findings on septal defects could be due to chance, and need replication.

## Background

Migraine is a common neurovascular disorder with a 1-year prevalence of 9%–22% in women and a peak prevalence during the reproductive years (Lipton et al., 2007). Improvement of migraine severity has been reported in 55%–90% of women during pregnancy (Banhidy et al., 2007). However, many women continue to suffer from migraine attacks during pregnancy and 7% have a first-onset migraine during pregnancy (Banhidy et al., 2007). Specific therapies for the acute treatment of migraine include ergot alkaloids (dihydroergotamine (DHE)) and selective serotonin 5-HT receptor agonists (triptans), and others. Studies have reported that up to 70% of pregnant women with migraine use antimigraine drugs but the frequency and type of medication use varies depending on country of residence, access to healthcare, and healthcare delivery (Harris et al., 2017; Nezvalova-Henriksen et al., 2009).

Ergotamine use during pregnancy increases sympathetic activity and vasoconstriction, leading to multiple adverse effects such as decreased uterine blood flow and increased uterine muscle contractility (Pertz et al., 1999). DHE, an ergot alkaloid, is also perceived to be associated with a high risk of adverse pregnancy events. However, DHE has significantly less vasoconstrictive and uterotonic effects compared to ergotamine (Pertz et al., 1999). Although chronic DHE exposure at supra-therapeutic levels during pregnancy in animal reproductive toxicology studies has been associated with delayed skeletal ossification and reduced birth weights, similar findings have not been reported in humans (Marchenko et al., 2015). The antimigraine activity of DHE mesylate is likely related to its agonist activity at 5-HT_1B_, 5-HT_1D_, and 5-HT_1F_ receptors leading to meningeal vasoconstriction and trigeminal inhibition (Silberstein et al., 2003; Dahlöf and Maassen Van Den Brink, 2012). A retrospective analysis by Bérard and Kori (Bérard and Kori, 2012) of DHE exposures among subjects within a large human pregnancy cohort found that DHE exposure did not statistically significantly increase the risk of low birth weight (LBW), major congenital malformations (MCM), or spontaneous abortions (SA); however, DHE exposure was associated with a four-fold increased risk of prematurity. More recently, in a population-based cohort of pregnant women from the province of Quebec in Canada, Bérard et al. (2021) have presented similar results but have also shown that triptans were associated with the risk of SA. Triptans are effective for the acute management of migraines and exert their effects by binding to the serotonin 5-HT receptors, thereby leading to vasoconstriction and inhibition of neuronal inflammation (Olesen et al., 2000). Based on the accumulated evidence from the sumatriptan pregnancy registry (Harris et al., 2017) and other studies (Amundsen et al., 2015; Afridi, 2020; Afridi, 2018), the risk of MCM was reported to be similar to the baseline risk in the general population (3%–5%) (Olesen et al., 2000). Similarly to Bérard et al. (2021), a meta-analysis showed that gestational use of triptans was not associated with the risk of MCM but did suggest an increased risk of SA (Marchenko et al., 2015). Dudman et al. (2022), in a 2021 meta-analysis have shown that untreated migraine was associated with some adverse pregnancy outcomes, and that triptans did not increase the risk of pregnancy outcomes when compared to the general population. They were not however able to assess other migraine medications.

Because migraine attacks are common during pregnancy, more data are needed to guide prescribers and help better characterize the relative benefits and potential risks, to both women and their fetuses, of therapies utilized for the acute treatment of migraine. In addition, since migraine treatment varies depending on access to healthcare and healthcare delivery, the aim of this study was to quantify the risk of prematurity, LBW, MCM, and SA associated with gestational use of DHE, and compare DHE and triptan use during pregnancy in terms of adverse pregnancy outcomes, in a cohort of pregnant women with private healthcare insurance in the US.

## Materials and Methods

### Database used

The US Merative Marketscan Research Database (Marketscan, 2011–2021), which includes data on a nationally representative sample of individuals with employer-sponsored health insurance in the United States (US), was used. The database encompasses demographic and insurance enrollment information, medical visits and hospitalizations, diagnoses and procedures received either as inpatients or outpatients, and prescriptions filled on an outpatient basis.

### Pregnancy-child cohort definition

Within the already collected data from the US MarketScan database, we have identified all completed pregnancies among women aged 18–45 years and linked these pregnancies to live-born infants using state, family identification numbers, and delivery and birth dates (for analyses on prematurity, low birth weight, and major congenital malformations). All pregnancies were considered for analyses on spontaneous abortion (SA).

Using a validated algorithm (Margulis et al., 2013), we estimated the date of the last menstrual period (first day of pregnancy) based on the delivery date, diagnostic codes indicating preterm delivery, or spontaneous abortion.

### Inclusion and exclusion criteria

Pregnant individuals were required to have continuous insurance coverage from 3 months prior to the first day of pregnancy until 6 months following the end of the pregnancy (delivery or SA). Newborns needed to be insured from birth until 6 months of age, unless they died sooner. Pregnancies exposed to known teratogenic medications during the first trimester and pregnancies with chromosomal abnormalities were excluded. This exclusion was based on the unlikelihood of chromosomal abnormalities being attributed to medication exposure. For SA analyses, we excluded women with planned abortions or those whose abortions occurred at less than 6 completed weeks of gestation (as these are potentially subject to misclassification due to the clinical non-recognition of many early pregnancy losses). We also excluded pregnancies with SA occurring after 22 weeks of gestation, as this is clinically defined as a premature birth.

### Study design

Cohort study. All pregnant individuals meeting inclusion and exclusion criteria were included in the study as the data were already collected and available in the Marketscan database; no sampling was done.

### Exposure to DHE and triptans

Exposure was defined as having filled at least one prescription for DHE, or triptan (almotriptan, eletriptan, naratriptan, rizatriptan, sumatriptan, and zolmitriptan), during pregnancy. Prescription fillings before pregnancy with durations overlapping with the beginning of gestation were also defined as a pregnancy exposure. When studying SA (see section on Outcomes in the next paragraph for the definition of SA), the exposure period of interest was defined as the beginning of pregnancy until the date of the SA or the corresponding index date for the comparators; for prematurity or LBW, the exposure period of interest was any time during pregnancy; and for MCM, the exposure period of interest was the first trimester of pregnancy (see section on Outcomes in the next paragraph for the definition of prematurity, LBW, MCM).

### Outcomes

MCM diagnosed in the first 6 months of life were identified with ICD-9 codes (740-759 excluding codes of minor congenital malformations or chromosomal abnormalities: 743.6, 744.1-744.4, 744.8, 744.9, 747.0, 747.5, 750.0, 752.4, 752.5, 754.6, 755.0, 755.1, 757.2-757.6, 757.8, 757.9, 758.4) and ICD-10 codes (Q00-Q99, excluding codes of minor malformations or chromosomal abnormalities: Q08-Q10, Q162, Q17-Q19, Q250, Q270, Q381, Q515, Q516, Q20-Q53, Q664-Q666, Q689, Q70, Q81-Q84, Q94-Q95). All organ systems were considered. This has been done in other studies with the US Merative Marketscan database (Kirby et al., 2014).

Prematurity was defined as a spontaneous delivery at <37th completed weeks’ gestation (Vilain et al., 2008). This has been used in other studies (Kirby et al., 2014) and has been validated before in the US IBM Marketscan database (Kirby et al., 2014).

LBW was defined as a newborn weighting <2,500 g (Vilain et al., 2008).

Spontaneous abortion (SA) (ICD-9-10 codes 630-634, 690.0 and 690.9) was defined has a pregnancy ending between the 6th and 22nd weeks’ gestation. Planned abortions were excluded from analyses.

### Statistical analyses

Four separate analyses were performed within the study cohort. MCM, prematurity and LBW were defined at delivery using the previously mentioned definitions, and using a traditional closed cohort design (Rothman and Lash, 2008).

For the analyses of spontaneous abortions, we performed a nested case–control design within the study cohort. Cases were defined as women with a diagnosis or a procedure for spontaneous abortion between the sixth and the 22nd week of gestation. The index date was defined as the calendar date of the spontaneous abortion. Because of our plan to assess several specific anti-migraine medications simultaneously, we randomly selected up to 5 controls for each case matched on gestational age and calendar year at the time of the event (spontaneous abortion or matched index date for selected controls). Similar to the methods of Einarson and colleagues (Einarson et al., 2009), we matched controls by the case’s index date and thus gestational age at the time of the spontaneous abortion. We did this because the risk of pregnancies ending in a loss is highly dependent on the gestational age at which the pregnancy is recognized, and because the probability of a spontaneous abortion being clinically detected increases with gestational age. Therefore, using a nested case–control design, we selected controls from among women who did not have a spontaneous abortion at or before the same gestational age as their matched case did. The index date of the controls was the same as that for the matched case. The nested case–control design gives similar effect sizes as the prospective cohort approach but has greater computational efficiency (Rothman and Lash, 2008; Essebag et al., 2003). Furthermore, this design for the study of spontaneous abortions has been used before in pregnancy studies (Bérard et al., 2021; Einarson et al., 2009; Nakhai-Pour et al., 2011; Nakhai-Pour et al., 2010).

Potential confounders considered for all analyses were known risk factors or associated with risk factors for the 4 studied outcomes (all these variables were either risk factors or determinants for adverse pregnancy outcomes): 1) sociodemographic variables including maternal age, employment status, and area of residence (urban/rural); 2) Maternal chronic co-morbidities including migraine to account for indication bias, hypertension, diabetes (Type I or II), asthma, depression, and thyroid disorders. The previous conditions were identified from either diagnoses or filled prescriptions of related medications; 3) smoking, alcohol of illicit drug use before or during pregnancy; 4) Healthcare utilization during the 3-months prior to pregnancy until the end of the critical time-window for the outcome studied including hospitalizations or emergency department (ED) visits (yes/no), visits to a specialist or general practitioner (yes/no); 5) Pregnancy related variables including previous pregnancy in the year before pregnancy, follow-up by an obstetrician, and use of high dose folic acid during pregnancy (>5 mg/day). We also considered whether other medications, including non-steroidal anti-inflammatory drugs (NSAIDs) or opioids, were used during pregnancy. Univariate and multivariate generalized estimation equation (GEE) models were built to quantify the independent association between the use of DHE, or triptans during pregnancy and the risk of prematurity, LBW, major congenital malformations, and spontaneous abortions adjusting for clinically important confounders and socioeconomic variables. The GEE models were also used to take into account inter-pregnancy variations as well as within-woman variations for those with multiple pregnancies between 2011-2021. Furthermore, the great advantage of GEE is that it provides parameter estimates and their (asymptotically) correct standard errors, and hence (asymptotically) correct inferences (tests, confidence intervals, etc.) even in cases when the correlation structure is not correctly specified (Pekár and Brabec, 2018). It can be used for cohort and case-control studies as well as nested-case-control design.

Risk ratios (RR; prematurity, LBW, MCM) and odds-ratios (OR; SA) with 95% confidence intervals (CI) were calculated using SAS System for Windows (SAS Institute Inc., North Carolina, United States). Differences were considered statistically significant when the 95% CIs did not overlap 1.0 and when P values (2-tailed) were less than 0.05.

### Ethics statement

The study was approved by the CHU Sainte-Justine Institutional Review Board, Montreal, Quebec, Canada (approval number, 2022-164). All personalized data were anonymized, and thus no patient consents were needed. The CHU Sainte-Justine Institutional Review Board took this into consideration when giving authorization as this is usually done in similar studies. Data were only accessible to the study team; data are hosted on a secure server at CHU Sainte-Justine.

## Results

Of the 767,994 pregnancies meeting inclusion criteria between 2011 and 2021, and considered for the analyses on prematurity, LBW, and MCM, 189 (0.02%)) were exposed to DHE, and 4,309 (0.56%) to triptans. All DHE exposures during pregnancy (n, 189) occurred in the first trimester, with prescription fillings before pregnancy with duration overlapping the first day of gestation, indicative of inadvertent exposures. For analyses on SA, 11,121 cases met our study definition, and 111,570 controls were sampled and matched on gestational age and calendar year of the event.

### Prematurity

For this analysis, 34,977 (4.6%) pregnant women had a premature delivery (Table 1); deliveries were mostly between 32- and 36-weeks’ gestation (34,937; 99.9%) (Table 2). Those with a preterm delivery were older; more likely to suffer from migraine; be smokers or using illicit drugs; had more hospitalizations, and used medications other than the ones studied here; they were also more likely to be followed by an obstetrician and take high dose folic acid (Table 1). Finally, those with a preterm delivery were more likely to be using opioids, be depressed, hypertensive and diabetics (Table 1). Adjusting for all potential confounding variables including maternal migraine, DHE (first trimester, adjusted RR 1.17, 95%CI 0.14, 9.74; 10 exposed cases), or triptan (adjusted RR 0.94, 95%CI 0.80, 1.10; 221 exposed cases), use during pregnancy were not statistically significantly associated with the risk of prematurity (Table 1).

**TABLE 1:** Association between DHE and triptan exposure during pregnancy and the risk of prematurity.

	Prematurity <37 completed weeks of gestation			
Characteristics	Yes n = 34,977	No n = 733,017	Crude RR (95%CI)	Adjusted RR (95%CI)
	n (%)			
Study medication exposure during pregnancy (yes/no) Dihydroergotamine Triptans^a^	10 (0.03) 221 (0.63)	179 (0.02) 4,088 (0.56)	1.49 (0.21-10.55) 1.06 (0.93-1.22)	1.17 (0.14-9.74) 0.94 (0.80-1.10)
Maternal age at the 1DG^d^ - mean ± SD	32.18 ± 4.65	31.87 ± 4.36	1.02 (1.01-1.02)	1.00 (1.00-1.01)
Employment Status Active Full Time Active Part Time or Seasonal Other/unknown	19,909 (56.92) 365 (1.04) 14,703 (42.04)	416,327 (56.80) 8,424 (1.15) 308,266 (42.05)	Ref. 0.91 (0.82-1.01) 1.00 (0.97-1.02)	Ref. 0.98 (0.88-1.09) 0.99 (0.97-1.01)
Urban dweller (yes/no)	31,380 (89.72)	660,583 (90.12)	0.96 (0.92-0.99)	0.91 (0.88-0.94)
Migraine diagnosis during pregnancy	627 (1.79)	11,354 (1.55)	1.16 (1.07-1.26)	1.12 (1.00-1.26)
Maternal comorbidities in the 3-month prior to 1DG^e^ Hypertension Diabetes Asthma Depression Thyroid disorders	5,061 (14.47) 3,183 (9.10) 3,017 (8.63) 4,691 (13.41) 3,034 (8.67)	42,017 (5.73) 35,902 (4.90) 56,218 (7.67) 80,136 (10.93) 50,766 (6.93)	2.76 (2.68-2.85) 1.94 (1.87-2.01) 1.14 (1.09-1.18) 1.26 (1.22-1.30) 1.28 (1.23-1.33)	2.02 (1.95-2.09) 1.05 (1.00-1.09) 0.98 (0.94-1.02) 1.07 (1.04-1.11) 1.03 (0.99-1.07)
Diagnosis of dependence to Tobacco Alcohol Other drugs	877 (2.51) 67 (0.19) 116 (0.33)	13,519 (1.84) 1,275 (0.17) 1,327 (0.18)	1.37 (1.27-1.46) 1.10 (0.86-1.41) 1.83 (1.51-2.21)	1.19 (1.11-1.28) 0.91 (0.71-1.18) 1.44 (1.17-1.76)
In the 3-months prior to the 1DG Hospitalization (yes/no) General practitioner visits - mean ± SD 0 1 2 or more Specialist visits - mean ± SD 0 1 2-4 5 or more Other medications^f^ - mean ± SD 0 1 2-3 4 or more	2,911 (8.32) 1.67 ± 3.73 17,355 (49.62) 6,270 (17.93) 11,352 (32.46) 5.33 ± 7.25 6,745 (19.28) 5,166 (14.77) 9,924 (28.37) 13,142 (37.57) 3.46 ± 3.65 9,552 (27.31) 4,010 (11.46) 7,518 (21.49) 13,897 (39.73)	62,540 (8.53) 1.41 ± 3.11 383,087 (52.26) 138,714 (18.92) 211,216 (28.81) 4.08 ± 5.68 155,749 (21.25) 129,505 (17.67) 226,656 (30.92) 221,107 (30.16) 2.86 ± 3.09 214,483 (29.26) 100,596 (13.72) 175,099 (23.89) 242,839 (33.13)	0.98 (0.94-1.01) 1.02 (1.02-1.02) Ref. 1.00 (0.97-1.03) 1.18 (1.16-1.21) 1.03 (1.03-1.03) Ref. 0.92 (0.89-0.96) 1.01 (0.98-1.04) 1.37 (1.33-1.41) 1.06 (1.05-1.06) Ref. 0.90 (0.86-0.93) 0.97 (0.94-1.00) 1.28 (1.25-1.32)	1.15 (1.07-1.24) Ref. 0.98 (0.88-1.09) 0.99 (0.97-1.01) Ref. 0.86 (0.82-0.89) 0.87 (0.84-0.90) 0.92 (0.89-0.95) Ref. 0.96 (0.92-1.00) 0.97 (0.94-1.00) 1.03 (1.01-1.07) NA NA NA
Exposure during the pregnancy (yes/no) NSAIDs^b^ Opioids^c^	1,055 (3.02) 3,258 (9.31)	16,767 (2.29) 56,386 (7.69)	1.23 (1.15-1.31) 1.19 (1.15-1.24)	1.05 (0.98-1.12) 1.01 (0.97-1.06)
Pregnancy follow-up by obstetrician	13,661 (39.06)	88,782 (12.11)	4.63 (4.53-4.74)	4.28 (4.18-4.39)
Pregnancy in the year prior the 1DG	2,033 (5.81)	50,749 (6.92)	0.83 (0.80-0.87)	0.69 (0.64-0.75)
High dose folic acid consumption before the end of the 1st trimester (>5 mg/day)	4,719 (13.49)	82,261 (11.22)	1.23 (1.20-1.27)	1.10 (1.07-1.14)

^a^ Including almotriptan, eletriptan, naratriptan, rizatriptan, sumatriptan, and zolmitriptan.

^b^ Including celecoxib, diclofenac, diflunisal, etodolac, fenoprophen, fentanyl, flurbiprofene, hydrocodone, hydromorphone, ibuprofen, indomethacin, ketoprofen, mefenamic acid, meloxicam, nabumetone, naproxen, piroxicam, rofecoxib, sulindac, tiaprofenic acid, tolmetin, and valdecoxib.

^c^ Including codeine, meperidine, morphine, oxaprozin, oxycodone, oxymorphone, pentazocine, tapentadol, propoxyphen, and tramadol.

^d^ First day of gestation defined as the first day of the last menstrual period.

^e^ Comorbidities were assessed in the 3 months prior to the 1DG using ICD-9 and ICD-10 diagnosis codes and prescribed medications.

^f^ Other pescribed medications than the study medications and the medications for the identification of the comorbidities.

**TABLE 2:** DHE and triptan exposure during pregnancy stratified by sub-categories of prematurity.

		Prematurity <37 completed weeks of gestation n = 34,977		
	Term pregnancies 37 or more completed weeks of gestation	Moderate or late preterm 32–36 weeks of gestation	Very preterm 28–31 weeks of gestation	Extreme preterm <28 weeks of gestation
	Yes n = 733,017	Yes n = 34,937	Yes n = 27	Yes n = 13
Study medication exposure	179 (0.02)	10 (0.03)	0 (0.0)	0 (0.0)
Dihydroergotamine Triptans^a^	4,088 (0.56)	220 (0.63)	1 (3.70)	0 (0.0)

^a^ Including almotriptan, eletriptan, naratriptan, rizatriptan, sumatriptan, and zolmitriptan.

### LBW

Within the study population, 2,545 (0.3%) pregnant women delivered a LBW newborn (Table 3). Those with a LBW newborn were older; more likely to be hypertensive, diabetics, or suffer from thyroid disorders; smokers, alcohol and illicit drug users; using NSAIDs; or using others medications concomitantly; followed by an OB/GYN, and using high dose folic acid (Table 3). Adjusting for potential confounders including maternal migraine, DHE (first trimester, adjusted RR 7.76, 95%CI 0.99, 60.83; 7 exposed cases), and triptans (adjusted RR 1.39, 95%CI 0.85, 2.27; 22 exposed cases), were not statistically significantly associated with the risk of LBW (Table 3), although estimates lacked statistical power.

**TABLE 3:** Association between DHE and triptan exposure during pregnancy and the risk of LBW.

	LBW <2,500 gr			
Characteristics	Yes n = 2,545	No n = 765,449	Crude RR (95% CI)	Adjusted RR (95% CI)
	n (%)			
Study medication exposure during the pregnancy (yes/no) Dihydroergotamine Triptans^a^	7 (0.27) 22 (0.86)	182 (0.02) 4,287 (0.56)	12.29 (1.21-124.83) 1.43 (0.93-2.18)	7.76 (0.99-60.83) 1.39 (0.85-2.27)
Maternal age at the 1DG^d^ - mean ± SD	32.19 ± 4.50	31.88 ± 4.37	1.02 (1.01-1.03)	1.00 (0.99-1.01)
Employment Status Active Full Time Active Part Time or Seasonal Other/unknown	1,713 (67.31) 35 (1.38) 797 (31.32)	434,523 (56.77) 8,754 (1.14) 322,172 (42.09)	Ref. 1.01 (0.73-1.42) 0.63 (0.58-0.96)	Ref. 1.07 (0.76-1.49) 0.64 (0.58-0.70)
Urban dweller (yes/no)	2,312 (90.84)	689,651 (90.10)	1.09 (0.95-1.25)	1.03 (0.90-1.18)
Migraine diagnosis during pregnancy	43 (1.69)	11,938 (1.56)	1.09 (0.80-1.47)	0.84 (0.60-1.16)
Maternal comorbidities in the 3-months prior to 1DG^e^ Hypertension Diabetes Asthma Depression Thyroid disorders	380 (14.93) 213 (8.37) 199 (7.82) 296 (11.63) 225 (8.84)	46,698 (6.10) 38,872 (5.08) 59,036 (7.71) 84,521 (11.04) 53,575 (7.00)	2.70 (2.42-3.01) 1.71 (1.48-1.96) 1.01 (0.88-1.17) 1.06 (0.94-1.20) 1.29 (1.12-1.48)	2.19 (1.95-2.47) 1.00 (0.86-1.16) 0.89 (0.77-1.03) 0.92 (0.81-1.04) 1.08 (0.94-1.24)
Diagnosis of dependence to Tobacco Alcohol Other drugs	68 (2.67) 10 (0.39) 14 (0.55)	14,328 (1.87) 1,332 (0.17) 1,429 (0.19)	1.44 (1.13-1.83) 2.26 (1.21-4.22) 2.96 (1.74-5.01)	1.40 (1.08-1.78) 2.02 (1.05-3.89) 2.35 (1.36-4.06)
In the 3-months prior to the 1DG Hospitalization (yes/no) General practitioner visits - mean ± SD 0 1 2 or more Specialist visits - mean ± SD 0 1 2-4 5 or more Other medications^f^ - mean ± SD 0 1 2-3 4 or more	158 (6.21) 1.35 ± 2.48 1,309 (51.43) 495 (19.45) 741 (29.12) 5.03 ± 6.76 482 (18.94) 398 (15.64) 758 (29.78) 907 (35.64) 3.49 ± 3.53 604 (23.73) 355 (13.95) 558 (21.93) 1,028 (40.39)	65,293 (8.53) 1.43 ± 3.14 399,133 (52.14) 144,489 (18.88) 221,827 (28.98) 4.14 ± 5.76 162,012 (21.17) 134,273 (17.54) 235,822 (30.81) 233,342 (30.48) 2.88 ± 3.12 223,431 (29.19) 104,251 (13.62) 182,059 (23.78) 255,708 (33.41)	0.71 (0.60-0.83) Ref. 1.04 (0.94-1.16) 1.02 (0.93-1.11) Ref. 1.00 (0.87-1.14) 1.08 (0.96-1.21) 1.31 (1.17-1.46) Ref. 1.26 (1.10-1.44) 1.13 (1.01-1.27) 1.49 (1.34-1.64) NA NA NA	0.67 (0.49-0.91) Ref. 1.02 (0.92-1.13) 0.93 (0.85-1.03) Ref. 0.91 (0.80-1.04) 0.94 (0.84-1.06) 0.97 (0.85-1.09) Ref. 1.14 (1.00-1.31) 1.00 (0.89-1.13) 1.14 (1.01-1.30) NA NA NA
Exposure during the pregnancy (yes/no) NSAIDs^b^ Opioids^c^	119 (4.68) 217 (8.53)	17,703 (2.31) 59,427 (7.76)	2.08 (1.71-2.53) 0.96 (0.83-1.11)	1.74 (1.43-2.11) 0.84 (0.72-0.97)
Pregnancy follow-up by obstetrician	834 (32.77)	101,609 (13.27)	3.18 (2.93-3.46)	2.88 (2.63-3.17)
Pregnancy in the year prior the 1DG	120 (4.72)	52,662 (6.88)	0.67 (0.56-0.80)	0.91 (0.65-1.29)
High dose folic acid consumption before the end of the 1st trimester (>5 mg/day)	342 (13.44)	86,638 (11.32)	1.22 (1.08-1.36)	1.05 (0.94-1.18)

a Including almotriptan, eletriptan, naratriptan, rizatriptan, sumatriptan, and zolmitriptan.

^b^ Including celecoxib, diclofenac, diflunisal, etodolac, fenoprophen, fentanyl, flurbiprofene, hydrocodone, hydromorphone, ibuprofen, indomethacin, ketoprofen, mefenamic acid, meloxicam, nabumetone, naproxen, piroxicam, rofecoxib, sulindac, tiaprofenic acid, tolmetin, and valdecoxib.

^c^ Including codeine, meperidine, morphine, oxaprozin, oxycodone, oxymorphone, pentazocine, tapentadol, propoxyphen, and tramadol.

^d^ First day of gestation defined as the first day of the last menstrual period.

^e^ Comorbidities were assessed in the 3 months prior to the 1DG, using ICD-9, and ICD-10, diagnosis codes and prescribed medications.

^f^ Other prescribed medications than the study medications and the medications for the identification of the comorbidities.

### Major congenital malformations (MCM)

In this study, 6,423 (1.0%) newborns had an MCM (Table 4). MCM was associated with increased maternal age; hypertension, diabetes, depression, thyroid disorders; smoking; use of other prescribed medications (other than the studied drugs) and opioids; and more specialist visits before pregnancy (Table 4). Women with a newborn with MCM were more likely to be followed by an OB/GYN, and use high dose folic acid (Table 4). Adjusting for confounders including maternal migraine, gestational exposure to DHE was not associated with a statistically significant risk of MCM (first trimester, adjusted RR 2.27, 95%CI 0.97, 5.29; 12 exposed cases); no increase in MCM was observed in users of triptans (adjusted RR 0.83, 95%CI 0.58, 1.19; 30 exposed cases) (Table 4). Among the 12 pregnant women exposed to DHE during pregnancy and with an infant diagnosed with MCM (Table 4), all were cardiac septal defects without further definition or diagnostic details (adjusted RR 6.62, 95%CI 1.00, 43.79); among these pregnant women, 2 were also exposed to diuretics and 2 to paroxetine, in addition to DHE during pregnancy). Triptan use was not associated with any risk of organ-specific malformations.

**TABLE 4:** Association between DHE and triptan exposure during the first trimester of pregnancy and the risk of major congenital malformation.

	Overall major congenital malformations			
Characteristics	Yes n = 6,423	No n = 319,537	Crude RR (95%CI)	Adjusted RR (95%CI)
	n (%)			
Exposure during the 1st trimester of pregnancy Dihydroergotamine Triptans^a^	12 (0.18) 30 (0.47)	177 (0.06) 1,508 (0.47)	2.91 (0.94-8.96) 0.97 (0.67-1.39)	2.27 (0.97-5.29) 0.83 (0.58-1.19)
Maternal age at the 1DG^d^ - mean ± SD	32,18 ± 4.46	32,05 ± 4.34	1.01 (1.00-1.01)	1.00 (0.99-1.00)
Employment Status Active Full Time Active Part Time or Seasonal Other/unknown	3,900 (60.72) 71 (1.11) 2,452 (38.18)	192,723 (60.31) 3,635 (1.14) 123,179 (38.55)	Ref. 0.96 (0.76-1.22) 0.98 (0.93-1.04)	Ref. 1.00 (0.79-1.26) 0.99 (0.93-1.04)
Urban dweller (yes/no)	5,795 (90.22)	288,258 (90.21)	1.00 (0.92-1.88)	0.97 (0.89-1.06)
Migraine diagnosis during pregnancy	124 (1.93)	4,913 (1.54)	1.26 (1.05-1.51)	1.14 (0.94-1.38)
Maternal comorbidities in the 3-months prior to 1DG^e^ Hypertension Diabetes Asthma Depression Thyroid disorders	535 (8.33) 423 (6.59) 531 (8.27) 764 (11.89) 507 (7.89)	19,343 (6.05) 16,311 (5.10) 24,755 (7.75) 35,214 (11.02) 22,909 (7.17)	1.41 (1.29-1.54) 1.31 (1.19-1.45) 1.07 (0.98-1.17) 1.09 (1.01-1.18) 1.11 (1.01-1.22)	1.23 (1.13-1.35) 1.03 (0.92-1.14) 1.00 (0.91-1.10) 1.01 (0.93-1.09) 1.00 (0.92-1.10)
Diagnosis of dependence to Tobacco Alcohol Other drugs	136 (2.12) 15 (0.23) 16 (0.25)	5,741 (1.80) 566 (0.18) 590 (0.18)	1.18 (1.00-1.40) 1.32 (0.79-2.21) 1.35 (0.82-2.22)	1.13 (0.95-1.34) 1.22 (0.72-2.07) 1.20 (0.72-1.99)
In the 3-months prior to the 1DG Hospitalization (yes/no) General practitioner visits - mean ± SD 0 1 2 or more Specialist visits - mean ± SD 0 1 2-4 5 or more Other medications^f^ - mean ± SD 0 1 2-3 4 or more	506 (7.88) 1.40 ± 2.76 3,329 (51.83) 1,183 (18.42) 1,911 (29.75) 4.75 ± 4.60 1,197 (18.64) 1,022 (15.91) 1,936 (30.14) 2,268 (35.31) 3.22 ± 3.38 1,720 (26.78) 836 (13.76) 1,452 (22.61) 2,415 (37.60)	27,647 (8.65) 1.43 ± 3.14 165,337 (51.74) 61,245 (19.17) 92,955 (29.09) 4.16 ± 4.14 66,249 (20.73) 56,521 (17.69) 99,168 (31.03) 97,599 (30.54) 2.94 ± 3.13 89,059 (27.87) 44,002 (13.77) 77,687 (24.31) 108,789 (34.05)	0.90 (0.82-0.99) 1.00 (0.99-1.00) Ref. 0.96 (0.90-1.03) 1.02 (0.96-1.08) 1.02 (1.01-1.02) Ref. 1.00 (0.92-1.09) 1.08 (1.00-1.16) 1.29 (1.20-1.30) 1.03 (1.02-1.04) Ref. 0.98 (0.91-1.07) 0.97 (0.90-1.04) 1.15 (1.08-1.22)	0.97 (0.81-1.16) Ref. 0.95 (0.89-1.02) 0.98 (0.92-1.04) Ref. 0.98 (0.90-1.07) 1.04 (0.96-1.12) 1.14 (1.06-1.24) Ref. 1.01 (0.92-1.10) 0.96 (0.90-1.04) 1.03 (0.95-1.12) NA NA NA
Exposure during pregnancy NSAIDs^b^ Opioids^c^	88 (1.37) 199 (3.10)	3,717 (1.16) 9,308 (2.91)	1.17 (0.94-1.45) 1.06 (0.90-1.21)	1.06 (0.90-1.24) 0.96 (0.88-1.06)
Pregnancy follow-up by obstetrician	1,416 (22.05)	42,127 (13.18)	1.86 (1.75-1.98)	1.75 (1.64-1.87)
Pregnancy in the year prior the 1DG	388 (6.04)	22,490 (7.04)	0.85 (0.77-0.94)	0.84 (0.69-1.02)
High dose folic acid consumption before the end of the 1st trimester (>5 mg/day)	783 (12.19)	36,124 (11.31)	1.09 (1.01-1.17)	1.04 (0.96-1.12)

^a^ Including almotriptan, eletriptan, naratriptan, rizatriptan, sumatriptan, and zolmitriptan.

^b^ Including celecoxib, diclofenac, diflunisal, etodolac, fenoprophen, fentanyl, flurbiprofene, hydrocodone, hydromorphone, ibuprofen, indomethacin, ketoprofen, mefenamic acid, meloxicam, nabumetone, naproxen, piroxicam, rofecoxib, sulindac, tiaprofenic acid, tolmetin, and valdecoxib.

^c^ Including codeine, meperidine, morphine, oxaprozin, oxycodone, oxymorphone, pentazocine, tapentadol, propoxyphen, and tramadol.

^d^ First day of gestation defined as the first day of the last menstrual period.

^e^ Comorbidities were assessed in the 3 months prior to the 1DG using ICD-9, and ICD-10, diagnosis codes and prescribed medications.

^f^ Other pescribed medications than the study medications and the medications for the identification of the comorbidities.

Note: The overall sample size decreases because we need at least 6 months of healthcare coverage after birth in order to identify MCM.

### Spontaneous abortions (SA)

Eleven thousand one hundred and twenty-one (11,121) cases of SA were identified, and 111,570 matched controls were analyzed (Table 5). Women with a SA were older; with no part-time or full-time employment; more likely to be hypertensive, diabetics, or had thyroid disorders; were smokers, drinking alcohol or used illicit drugs; more likely to be hospitalized; having physician visits; and followed by an OB/GYN (Table 5). They were also more likely to use NSAIDs, opioids or other medications, and high dose folic acid as well as having had another pregnancy in the year prior to this current pregnancy (Table 5). Adjusting for potential confounders including maternal migraine, DHE exposure during pregnancy was more than tripling the risk of SA but the estimate was statistically non-significant (first trimester, adjusted OR 3.19, 95%CI 0.98, 10.38; 23 exposed cases); triptan use during pregnancy was not associated with the risk of SA (adjusted OR 0.88, 95%CI 0.63, 1.24; 53 exposed cases) (Table 5).

**TABLE 5:** Association between DHE and triptan exposure during pregnancy and the risk of spontaneous abortion.

	Spontaneous abortion			
Characteristics	Cases n = 11,121	Matched controls n = 111,570	Crude OR (95% CI)	Adjusted OR (95% CI)
	n (%)			
Study medication exposure during the pregnancy (yes/no) Dihydroergotamine Triptans^a^	23 (0.21) 53 (0.48)	167 (0.15) 555 (0.50)	4.22 (1.01-17.62) 0.70 (0.50-0.97)	3.19 (0.98-10.38) 0.88 (0.63-1.24)
Maternal age at the 1DG^d^ - mean ± SD	31.31 ± 6.31	30.80 ± 6.04	1.01 (1.01-1.02)	1.00 (1.00-1.01)
Employment Status Active Full Time Active Part Time or Seasonal Other/unknown	6,034 (54.26) 131 (1.18) 4,956 (44.56)	62,457 (55.98) 1,236 (1.11) 47,877 (42.91)	Ref. 1.10 (0.92-1.30) 1.06 (1.02-1.10)	Ref. 1.11 (0.88-1.39) 1.08 (1.03-1.13)
Urban dweller (yes/no)	9,935 (89.34)	98,555 (88.33)	1.10 (1.03-1.17)	1.01 (0.94-1.08)
Migraine diagnosis during pregnancy	119 (1.07)	1,236 (1.11)	0.98 (0.82-1.17)	0.79 (0.64-1.01)
Maternal comorbidities in the 3-months prior to 1DG^e^ Hypertension Diabetes Asthma Depression Thyroid disorders	1,565 (14.07) 888 (7.98) 1,170 (10.52) 1,607 (14.45) 778 (7.00)	7,424 (6.65) 4,416 (3.96) 12,215 (10.95) 16,460 (14.75) 6,625 (5.94)	2.21 (2.08-2.34) 2.02 (1.88-2.18) 0.96 (0.90-1.02) 0.98 (0.93-1.03) 1.17 (1.09-1.27)	1.91 (1.73-2.11) 1.63 (1.50-1.77) 0.91 (0.83-0.99) 0.81 (0.76-0.87) 0.92 (0.83-1.02)
Diagnosis of dependence to Tobacco Alcohol Other drugs	452 (4.06) 57 (0.51) 111 (1.00)	1,861 (1.67) 355 (0.32) 496 (0.44)	2.37 (2.14-2.62) 1.59 (1.21-2.09) 2.13 (1.73-2.62)	2.20 (1.95-2.48) 1.12 (0.82-1.54) 1.78 (1.40-2.27)
In the 3-months prior to the 1DG Hospitalization (yes/no) General practitioner visits - mean ± SD 0 1 or more Specialist visits - mean ± SD 0 1 2-3 4 or more Other medications^f^ - mean ± SD 0 1 2-3 4 or more	1,280 (11.51) 1.59 ± 3.24 5,714 (51.38) 5,407 (48.62) 5.68 ± 7.46 2,218 (19.94) 1,358 (12.21) 3,000 (26.98) 4,545 (40.87) 3.36 ± 3.74 3,450 (31.02) 1,151 (10.35) 2,212 (19.89) 4,308 (38.74)	9,876 (8.85) 1.52 ± 3.48 58,431 (52.37) 53,139 (47.63) 4.12 ± 6.08 27,357 (24.52) 18,392 (16.48) 32,309 (28.96) 33,512 (30.04) 2.95 ± 3.28 34,424 (30.85) 13,901 (12.46) 25,052 (22.45) 38,193 (34.23)	1.29 (1.21-1.38) 1.01 (1.00-1.01) Ref. 1.04 (1.00-1.0) 1.03 (1.03-1.03) Ref. 0.92 (0.86-0.98) 1.14 (1.08-1.21) 1.64 (1.56-1.73) 1.03 (1.03-1.04) Ref. 0.84 (0.78-0.90) 0.89 (0.85-0.94) 1.12 (1.07-1.17)	1.11 (0.93-1.33) Ref. 1.06 (1.00-1.12) Ref. 0.86 (0.79-0.94) 1.01 (0.93-1.09) 1.21 (1.13-1.30) Ref. 0.86 (0.67-1.10) 0.80 (0.64-1.00) 0.80 (0.62-1.03) NA NA NA
Exposure during the pregnancy (yes/no) NSAIDs^b^ Opioids^c^	639 (5.75) 1,242 (11.17)	2,116 (1.90) 5,402 (4.84)	2.20 (1.99-2.43) 1.99 (1.85-2.14)	2.21 (1.99-2.45) 1.94 (1.79-2.10)
Pregnancy follow-up by obstetrician	7,431 (66.82)	44,972 (40.31)	2.62 (2.47-2.78)	2.62 (2.44-2.81)
Pregnancy in the year prior the 1DG	817 (7.35)	6,516 (5.84)	1.24 (1.15-1.33)	0.96 (0.77-1.19)
High dose folic acid intake before the end of the 1st trimester (>5 mg/day)	1,333 (11.99)	8,814 (7.90)	1.54 (1.45-1.64)	1.40 (1.28-1.53)

^a^ Including almotriptan, eletriptan, naratriptan, rizatriptan, sumatriptan, and zolmitriptan.

^b^ Including celecoxib, diclofenac, diflunisal, etodolac, fenoprophen, fentanyl, flurbiprofene, hydrocodone, hydromorphone, ibuprofen, indomethacin, ketoprofen, mefenamic acid, meloxicam, nabumetone, naproxen, piroxicam, rofecoxib, sulindac, tiaprofenic acid, tolmetin, and valdecoxib.

^c^ Including codeine, meperidine, morphine, oxaprozin, oxycodone, oxymorphone, pentazocine, tapentadol, propoxyphen, and tramadol.

^d^ First day of gestation defined as the first day of the last menstrual period.

^e^ Comorbidities were assessed in the 3 months prior to the 1DG using ICD-9, and ICD-10, diagnosis codes and prescribed medications.

^f^ Other prescribed medications than the study medications and the medications for the identification of the comorbidities.

## Discussion

In this privately insured US pregnancy cohort, first trimester DHE use for the treatment of acute migraine during pregnancy tended to increase the risk of LBW, SA and uncategorized cardiac septal defects, although the estimates were statistically non-significant. Triptan use was not associated with any of the studied outcomes. To our knowledge, this is the first study assessing the risk of DHE exposure during pregnancy in the US population. Although the study cohort was large, the prevalence of DHE and triptan use was low, and thus some estimates are unstable and lack statistical power.

We have found no association between DHE and prematurity comparable to results from Kallen et al. (Kallen et al., 2011), which utilized data from the Swedish Medical Birth Register. However, Bérard et al. (2021) and Bérard and Kori (Bérard and Kori, 2012) have found an increase in the risk of prematurity associated with gestational exposure to DHE, which could partly be explained by the fact that DHE exposure in our current study mostly occurred during the first trimester, whereas it was at the end of pregnancy in Bérard et al. (2021) This is important because the time-window of interest for prematurity is the second and third trimesters; this is even more relevant given that DHE can induce uterine contractions, which can result in preterm delivery when used later in gestation (Altman et al., 1952). Our findings on DHE and LBW are comparable to Kallen et al. (Kallen et al., 2011), Bérard and Kori (Bérard and Kori, 2012), Bérard et al. (2021), and Bánhidy et al. (2007), although our estimate lacks statistical power.

Our findings on DHE and MCM are somewhat similar to Bánhidy et al. (2007), and Bérard et al. (2021) Although DHE exposure during the first trimester of pregnancy suggested potential increased risk of uncategorized cardiac septal defects, our study is the first to show such an association, which could be due to chance finding. Although it is known that studying overall MCM as a category could mask some associations with specific organ systems (Fitzmaurice, 2000), our finding on septal defect needs to be replicated in further studies. Moreover, several newborns with septal defects were concomitantly exposed to diuretics or paroxetine *in utero*, which are known to increase the risk of congenital malformations, especially cardiac defects (Bar-Oz et al., 2007; Ueland, 1977). Hence, even if we have adjusted for concomitant medication use during pregnancy, residual confounding could remain. Furthermore, it should also be noted that septal defects can be transient and usually require a second confirmatory diagnosis, which could not be assessed in this study during the 6-months follow-up after birth compared to the 12-months follow-up in Bérard et al. (2021) Therefore, our unspecified categorization would have captured both atrial septal defects (most of which are patent foramen ovale, a normal finding affecting approximately 25% of newborns (Kheiwa et al., 2020)) as well as heterogenous ventricular septal defects, potentially leading to misclassification of outcome. The difference in follow-up periods between Bérard and Kori (2012), Bérard et al. (2021) and our current study is due to the fact that the US cohort was comprised of privately insured women/children who often switched to other health insurance plans after delivery/birth and longitudinal follow up becomes an issue. In contrast longer follow-ups were less of a problem in Quebec, Canada where healthcare coverage is universal. Lastly, toxicology studies performed on rats and rabbits exposed to supratherapeutic doses of oral and intranasal DHE showed no malformations related to the heart (Sassine et al., 1971).

Our findings on SA and DHE are also contrary to Bérard et al. (2021). This could partly be explained by different reporting and coding of SA in the US and Quebec, Canada databases, leading to misclassification of outcome. This is unlikely to occur for outcomes measured in-hospital such as prematurity or birth weight.

Our results on triptans are consistent with the literature. Indeed, we did not find that triptan use during pregnancy was increasing the risk of prematurity, LBW, and MCM similarly to Bérard et al. (2021) in Quebec, and other studies from Norway and Sweden (Kallen et al., 2011; Amundsen et al., 2016). Our lack of association for SA is also consistent with two meta-analyses (Marchenko et al., 2015; Dudman et al., 2022) but is contrary to Bérard et al. (2021) who found that triptans were increasing the risk of SA. This could again be partly explained by different reporting and categorization of SA between studies.

### Strengths and limitations

This large population-based study enabled us to evaluate the effect of acute-treatment-of-migraine drug use during pregnancy. Data on filled prescriptions do not rely on maternal recall. Diagnoses of major malformations as well as data on birth weight have been validated against patient charts in other studies (Vilain et al., 2008). Only clinically detected spontaneous abortions were considered, without relying on maternal recall. Spontaneous abortions that were never detected by the woman themselves were excluded, as was done in all other similar studies to date (Nakhai-Pour et al., 2011; Nakhai-Pour et al., 2010; Li et al., 2003). If DHE or triptans increase the risk of spontaneous abortions that are not clinically detected, our findings are conservative and thus would underestimate the true risk. However, if they are not associated with non-clinically detected spontaneous abortions, there is no reason to believe that misclassification would be different between cases and controls, resulting in non-differential misclassification. We adjusted our findings for maternal migraine, hence limiting potential confounding by indication. We also studied specific and acute-treatment-migraine therapeutics within a single population-based pregnancy cohort in the US, which considered migraine severity, and use of concomitant or complementary therapeutics during gestation. Therefore, we are confident that residual confounding by indication, if present, would not completely explain our findings. The evaluation of exposure, although validated, was based on filled prescriptions and might not necessarily reflect actual intake. However, we hypothesize that women who filled a prescription for a DHE or triptans took at least one dose as was done in other studies (Bérard and Kori, 2012; Bérard et al., 2021).

Within the US Merative Marketscan Research Database, information on potential confounding variables such as maternal obesity and over-the-counter (OTC) folic acid use are not exhaustively available. However, we have previously shown that maternal weight and pre-conceptual OTC folic acid intake are not strong enough confounders to reverse findings of associations between drug use during gestation and adverse pregnancy outcomes when adjusting for indication and healthcare utilization use as was done here (Bérard et al., 2009). Although maternal weight has been shown to be associated with the risk of MCM (Nezvalova-Henriksen et al., 2009), we doubt that women using DHE or triptan would differ significantly in terms of their weight, and thus this would not explain the differences in our risk estimates because maternal weight would not be considered a confounder. We have taken into account prescribed folic acid use, hence prescribed OTC use, and high dose folic acid use, which requires a prescription. Given that high dose folic acid is given to high-risk pregnancies, our estimates are likely markers of severity.

Although migraine was not statistically significantly increasing the risk of adverse pregnancy outcomes in our study (other than for prematurity), we cannot completely exclude the possibility of residual confounding by underlying disease in the risk estimates for DHE or triptans. Furthermore, our prevalence of MCM (1%) was lower than what has been reported in the literature (3%) (Moorthie et al., 2018). This could be explained in part by the short time period after birth where assessments were made, and thus we cannot completely rule out misclassification for this study outcome. Also, we cannot rule out the possibility of chance findings in 5% of our statistically significant associations. Furthermore, analyses of specific adverse pregnancy outcomes might have missed significant associations due to lack of statistical power, and estimates could be unstable given the small number of pregnant women taking DHE, or a triptan.

Finally, our study population was of higher socio-economic status (SES) than other studies published thus far in the literature on migraine treatment in pregnancy (Bérard et al., 2021; Kallen et al., 2011). Indeed, we only considered pregnant women insured by private insurance in the US. This could also partly explain differences between our study findings and others since SES could differently impact access and availability to drugs in pregnancy as well as pregnancy outcomes.

## Conclusion

After considering maternal migraine and concomitant migraine medication use, first trimester DHE use was not statistically significantly associated with an increased risk for prematurity, LBW, MCM, or SA; our findings on cardiac septal defect could be due to chance and need to be replicated. Additionally, no increased risk was observed for gestational exposure to triptans anytime during pregnancy. Even with 10 years of data available within a US privately insured cohort of pregnant women, it remains that results for DHE and triptans could be partly explained by their small number of exposed cases. Given that a recent study showed that almost 9 out of 10 women reported deliberate non-adherence to needed antimigraine medications during pregnancy out of fear of harming their unborn children (Amundsen et al., 2019), there is need for more publicly available and consistent information regarding the potential risks of antimigraine medication use during pregnancy.

## Data Availability

The data analyzed in this study is subject to the following licenses/restrictions: The use of the Marketscan databases (and thus data) was approved and provided under the condition that it is not made available publicly. Health data that are included in the database are sensitive and under the licencing agreement with Merative, the holder of Marketscan, hence we are not allowed to make crude data available. However, all aggregate data are included directly in the manuscript results. Requests to access these datasets should be directed to https://www.merative.com/documents/brief/marketscan-explainer-general.
